# Fe_2_O_3_ nanoparticles disrupt microstructure and reduce the viscoelasticity of simulated asthma airway mucus for potential airway mucus clearance applications

**DOI:** 10.3389/fphys.2025.1566716

**Published:** 2025-06-30

**Authors:** Jiayuan Zhong, Lei Shi, Zhiwei Liu, Kai Ni, Lei Liu, Yan Pan, Jingjing Li, Xiaowei Yu, Linhong Deng, Mingzhi Luo

**Affiliations:** ^1^ Changzhou Key Laboratory of Respiratory Medical Engineering, Institute of Biomedical Engineering and Health Sciences, School of Medical and Health Engineering, Changzhou University, Changzhou, China; ^2^ Department of Respiratory and Critical Care Medicine, The Affiliated Changzhou No. 2 People’s Hospital of Nanjing Medical University, Changzhou, China; ^3^ Wenzhou Key Laboratory of Biomaterials and Engineering, Wenzhou Institute, University of Chinese Academy of Sciences, Wenzhou, China

**Keywords:** asthma, airway mucus, rheology, Fe_2_O_3_ nanoparticles, expectorant agent

## Abstract

Fe_2_O_3_ nanoparticles have been developed as carriers to transport drugs through airway mucus (AM); however, their impacts on the rheological properties of AM, especially in disease states, are unknown. In this study, we investigated the abilities of Fe_2_O_3_ nanoparticles dispersed in various media to alter the microstructure and rheological behaviors of simulated asthmatic AM. Here, the simulated AM was prepared via reconstituted mucins and other components in a composition resembling that of human AM reported in asthma, followed by treatment with Fe_2_O_3_ nanoparticles before and after curing. Subsequently, the AM samples treated with and without Fe_2_O_3_ nanoparticles were examined for their microstructures by optical immunofluorescence microscopy and for the rheological behaviors via steady-state and dynamic rotational rheometry. The results indicate that the Fe_2_O_3_ nanoparticles disrupt the mucus microstructure by inducing protein aggregation to increase the pore size and fiber diameter of the AM. However, the Fe_2_O_3_ nanoparticles significantly reduced the magnitudes of the viscoelastic properties of AM, including apparent viscosity, yield stress, and dynamic viscoelastic modulus. Although the addition of Fe_2_O_3_ nanoparticles before and after curing of AM appeared to produce similar effects, these effects had greater magnitudes when the nanoparticles were added before curing. The effects were also dependent on the concentration and surface property determined by the dispersion medium of the nanoparticles; accordingly, Fe_2_O_3_ nanoparticles dispersed at a concentration of 0.4 mg/mL in H_2_O were the most potent at altering the microstructure and rheology of AM, producing better results than the concentration of 0.4 mg/mL of the conventional mucolytic chymotrypsin. Furthermore, tests on mucus samples collected from asthmatic patients showed similar results to those obtained with the simulated AM. Together, these findings suggest that Fe_2_O_3_ nanoparticles per se are useful as not only drug carriers but also expectorant agents for AM clearance therapy; they may also be more beneficial than pharmaceutical mucolytics owing to their wide availability and high biocompatibility.

## 1 Introduction

In healthy subjects, airway mucus (AM) acts as the first defense barrier of the respiratory system that captures and transports inhaled harmful pathogens into and from the airways; these actions are largely dependent on the rheological properties of the AM, ciliary movements, and cough-generated forces (Duncan et al., 2016; Papadopoulou et al., 2023; Covert et al., 2012). However, in airway diseases such as severe asthma, the AM is characterized by high viscoelasticity and poor fluidity that make it very difficult to expel. This may lead to AM embolism, respiratory failure, and even death of the patient (Nordgård et al., 2014; Mummy et al., 2022). Therefore, therapeutic reduction of the viscoelasticity of AM is essential in the treatment of airway diseases. In the past, both pharmacological (mucolytics) and physical interventions have been used in the clinic to help clear AM in asthmatic patients (Poole and Black, 2003). For example, N-acetylcysteine, which is a classical antioxidant, has been shown to be capable of reducing the viscoelasticity and thereby promoting the clearance of AM. Nonetheless, AM retention remains an unsolved problem in severe asthma (Krings and Gierada, 2023). Hence, there is great significance to developing alternative methods for effective reduction of the viscoelasticity of asthmatic AM.

The viscoelasticity of AM is primarily determined by mucins, which consist of a protein core with regions rich in serine and threonine, that enable a high grade of O-glycosylation (Ahn et al., 2015; Hill et al., 2022; Kavishvar and Ramachandran, 2023). Moreover, mucins are a family of glycosylated proteins with high molecular weights (10–40 MDa) that are secreted by goblet cells and submucosal glands. Once secreted, mucins experience multiple levels of assembly and curing, finally forming a unique network depending on covalent (disulfide bonds and Ca^2+^-mediated links) and non-covalent (hydrogen bonding and hydrophobic) interactions (Lai et al., 2009a). These specific structures make them capable of holding water and support the hydrogel-like complex viscoelastic behaviors (Carlson et al., 2018; Bansil and Turner, 2006; Yang et al., 2022; Meldrum et al., 2018; Argüeso and Gipson, 2001). The viscoelasticity of AM can be further modulated by external factors such as nanoparticles (Wagner et al., 2023). In fact, various nanoparticles have been studied for their abilities to penetrate and transport substances in AM as drug-delivery vehicles (Kirch et al., 2012; Hyun et al., 2008; Schuster et al., 2013). Among these, Fe_2_O_3_ nanoparticles have shown high efficiencies for drug encapsulation and transportation through AM (Witten and Ribbeck, 2017; Witten et al., 2018). Importantly, the small size of Fe_2_O_3_ nanoparticles makes them suitable for *in vivo* use as they can be removed through extravasation and renal clearance (Gupta and Wells, 2004). Previous studies have also shown that the presence of nanoparticles can lead to decreased viscosity of polymer solutions (Negrini et al., 2017; Goodwin et al., 2013; Mackay et al., 2003). However, it remains unknown whether Fe_2_O_3_ nanoparticles can reduce the viscoelasticity of AM, especially under asthmatic conditions.

Recently, polymer solutions prepared with reconstituted mucins and other components of AM at concentrations similar to those found in asthma patients have been shown to exhibit microstructures, biochemical responses, and bulk rheological properties close to those of real mucus from asthmatic patients; these enable their use as simulated asthmatic AM while offering experimental advantages like large sample availability and reduced sample heterogeneity (Liu et al., 2024; Lafforgue et al., 2017). Based on these advantages, we examined the effects of Fe_2_O_3_ nanoparticles on the microstructures and rheological properties of simulated AM through immunofluorescent microscopy as well as rotational and oscillatory rheometry. The results show that Fe_2_O_3_ nanoparticles considerably disrupt the microstructure and reduce the rheological properties of AM, including viscosity, yield stress, and viscoelastic modulus. Further tests with real mucus samples collected from asthmatic patients showed similar results to those of the simulated AM. Together, these findings suggest that Fe_2_O_3_ nanoparticles are indeed capable of reducing the viscoelasticity of the simulated asthmatic AM, most likely through disruption of mucin polymerization, and may hence have great potential to be developed into novel mucolytics for therapeutic clearance of AM in severe asthma.

## 2 Materials and methods

### 2.1 Materials

Mucin was purchased from Sigma-Aldrich (St. Louis, MO, United States; cat. no. M2378); bovine serum albumin (BSA) was purchased from BioFroxx (Guangzhou, China; cat. no. 4240); glutaraldehyde was obtained from Sinopharm Chemical Reagent Co., Ltd. (Shanghai, China; cat. no. G105905); phosphatidylcholine (PC; cat. no. L639160) and Fe_2_O_3_ nanoparticles (cat. no. F299311) were purchased from Aladdin (Shanghai, China); MUC5AC (cat. no. MA5-12178) primary antibody and goat anti-mouse IgG1 cross-adsorbed secondary antibody Alexa FluorTM 488 (cat. no. A21121) were sourced from Thermo Fisher Scientific (Waltham, MA, United States); chymotrypsin was procured from Macklin Biochemical Technology Co., Ltd. (Shanghai, China; cat. no. C804761); 4% paraformaldehyde was purchased from Yonghua Chemical Co., Ltd. (Suzhou, China; cat. no. 30525-89-4); dio was purchased from Beyotime Biotech Inc. (Shanghai, China; cat. no. C1038); FITC-BSA was purchased from Solarbio (Beijing, China; cat. no. SF063).

### 2.2 Preparation of simulated asthmatic AM

Simulated asthmatic AM was prepared using the mucins and other components according to the protocols described in literature (Liu et al., 2024; Hamed and Fiegel, 2013). Briefly, 6% mucin, 1% BSA, 1% PC, and 10% glutaraldehyde solution (crosslinking agent) were dissolved in phosphate-buffered saline (PBS) and rotated upside down for 6 d at a speed of 5 r/min (4°C) on a vertical mixer (HS-3, Xinzhi, China) for curing (cross-linking) and homogenization before usage (Thornton and Sheehan, 2004).

### 2.3 Preparation and characterization of Fe_2_O_3_ nanoparticles

Fe_2_O_3_ nanoparticles suspended in H_2_O, 1% PC, or 1% BSA at a stock concentration of 2 mg/mL were dispersed for 20 min using an ultrasonic cleaner (KQ10-300DTD, Zihua, China) and stored at 4°C. Before being used in the experiments, the Fe_2_O_3_ nanoparticle suspension was again treated with ultrasound waves for 10 min. The Fe_2_O_3_ nanoparticles in suspension were characterized for morphology and size via field-emission scanning electron microscopy (SUPRA55, Zeiss, Germany) as well as a nanolaser particle size and zeta potential analyzer (ZEN3600, Malvern, United Kingdom), respectively.

### 2.4 Treatment of AM with Fe_2_O_3_ nanoparticles or mucolytics

For experiments, the stock suspension of dispersed Fe_2_O_3_ nanoparticles was diluted to final concentrations of 0.03, 0.3, 0.4, 0.5, and 0.6 mg/mL. Then, an aliquot of the dispersed Fe_2_O_3_ nanoparticles at each final concentration was added to the aforementioned preparation of AM before or after addition of the 10% glutaraldehyde solution for curing over 6 d. If the Fe_2_O_3_ nanoparticles were introduced before adding the 10% glutaraldehyde solution, the Fe_2_O_3_ nanoparticle treatment was considered to target curing AM; otherwise, the treatment was considered to target cured AM. Simulated AM prepared in the same manner but combined with an aliquot of the dispersion medium without Fe_2_O_3_ nanoparticles (vehicle) was used as the control. To compare the effects of the Fe_2_O_3_ nanoparticles on the rheology of AM with those of a conventional mucolytic like chymotrypsin, we treated cured AM samples with 0.4 mg/mL of Fe_2_O_3_ nanoparticles and 0.4 mg/mL of chymotrypsin for 24 h before measuring the rheological behaviors.

### 2.5 Examination and characterization of AM microstructure

The microstructures of AM treated with and without Fe_2_O_3_ nanoparticles were examined and characterized by immunofluorescence microscopy. The AM sample was first sealed in an embedding medium for cryotomy (Tissue-Tek O.C.T. Compound, SAKURA, United States; cat. no. 4583) and cut into 8--μm-thin slices in a −25°C environment using a freezing microtome (Leica CM 1950, Leica Biosystems, Deer Park, IL, United States). The slices were then placed on glass slides and allowed to adhere at room temperature for 15 min before being fixed with 1 mL of 4% paraformaldehyde for 15 min. Thereafter, the slides were washed thrice with PBS to remove any residual paraformaldehyde, treated with NaBH_4_ for 5 min, and incubated with the MUC5AC primary antibody as well as goat anti-mouse IgG1 cross-adsorbed secondary antibody Alexa FluorTM 488 for 1 h at room temperature. After washing thrice with PBS, the slides were stained with Ponceau S (Solarbio, Beijing, China; cat. no. G1346) for 10 min and washed twice with PBS for 5 min each time. Subsequently, the slides were examined for the AM microstructure via fluorescence and bright-field microscopy using an inverted optical microscope (×40 objective, Axio Observer Z1, Zeiss, Germany); this microstructure was then imaged and quantitatively analyzed using ImageJ software (National Institutes of Health, Bethesda, MD, United States). The images were first converted to ImageJ-compatible 8-bit file format; then, the images were processed and analyzed with the StarDist program in the ImageJ Plugins option to recognize and quantify the microstructure pattern, pore area, and fiber diameter.

### 2.6 Rheological measurements on AM

The rheological measurements were performed using a rotational rheometer (Kinexus Pro, Malvern, United Kingdom) with the cone-and-plate geometry (cone diameter: 40 mm, gap: 1 mm, angle: 4 rad) according to a method reported previously (Liu et al., 2024). Briefly, a small amount of AM (∼1 mL) was loaded onto the plate by gentle pouring to minimize the shear effects. A thin layer of silicone oil was then applied to prevent dehydration and ensure a water-saturated atmosphere around the sample. The geometry was then lowered to the gap measurement, and the desired temperature (37°C) was set. Before commencing the experiment, each sample was presheared at a constant shear rate of 5 s^−1^ for 30 s and allowed to rest for 5 min thereafter for equilibration to obtain stable and reproducible measurements. The apparent viscosity and apparent yield stress of the AM were measured via the steady-state shear stress test by sweeping the shear rate from 0.01 to 100 s^−1,^ while the shear stress was ramped from 0.01 to 100 Pa. The thixotropy of the AM sample was quantified via the three-interval thixotropy test with stepwise changes to the shear rate to monitor the initial structure, break-up, and final recovery of the fluid sample successively. Briefly, the sample was first subjected to a very low shear condition of 0.01 s^–1^ for 30 s, which provided a reference for the fluid structure “at rest” or at least under very low shearing. Then, a higher shear condition of 100 s^–1^ for 30 s was imposed to disrupt the internal structure of the fluid at rest or with low shear; this applied shear rate of 100 s^−1^ was intended to represent the shearing of mucus due to cough. Lastly, the sample was allowed to recover under a very low shear condition of 0.01 s^–1^ for 600 s to test for changes in viscosity with shear rate and analyze changes in the thixotropic ring area of the AM.

The stress dependency response of the AM was measured through the stress oscillatory shear test at a constant frequency of 1 Hz; this is the classical test for determining the limit of the linear viscoelastic (LVE) range of a fluid. The results were interpreted based on evolutions of the elastic and viscous moduli (G′ and G″) as well as the loss angle (δ) as functions of a sinusoidal input. The strain dependency response of the AM was measured by the strain oscillatory shear test at a constant frequency of 1 Hz, while the strain amplitude was swept from 0.01% to 100% to obtain the LVE and non-LVE ranges. At each strain amplitude, 20 cycles of raw data were recorded for the torque and displacement at a sampling rate of 1,024 points per cycle. Based on the test results, 0.5% strain was determined to be within the LVE range and was thus used for the yield stress, creep-discovery, and frequency oscillatory shear tests. The lowest strain value used in the experiments was 10^−2^ or 0.01%, which is quite far from the lower detection limit of the rheometer (∼10^−8^%, Kinexus Pro, Malvern, United Kingdom). However, the results at a lower range of strain values (10^−2^%–10^−1^%) fluctuated considerably. Therefore, we only considered results in the strain range of 10^0^–10^2^ to evaluate the experimental effects. The frequency-dependent response of the AM was measured by the frequency oscillatory shear test at a constant strain amplitude (0.5% strain for the LVE range in this case) as the frequency was swept from 10^–2^ to 10^2^ Hz. The obtained LVE data for the frequency-dependent elastic modulus (G′) and viscous modulus (G″) were the in-phase and out-of-phase components of stress induced in the material divided by the applied strain magnitude, respectively. The tests were performed at small strain amplitudes to minimize the shear damage to the mucus sample.

### 2.7 Collection of mucus samples from asthmatic patients

Mucus samples were collected from six asthmatic patients at the Affiliated Changzhou No. 2 People’s Hospital of Nanjing Medical University, Changzhou, China. All patients gave their informed consent, and their personal clinical information was maintained anonymous and confidential. The study was approved by the Research Ethics Committee of Changzhou University (#2022022804, Changzhou, China). All procedures were performed in abeyance with the relevant guidelines and regulations. Mucus was induced by autogenic drainage and immediately transferred into sterile tubes. All collected samples were exposed to ultraviolet light for 1 h for sterilization. Then, the mucus samples were homogenized by vortexing and divided into three aliquots for the tests as control, Fe_2_O_3_ nanoparticles, and mucolytics (chymotrypsin). The rheological experiments conducted with the mucus samples from the asthmatic patients were identical to those performed with the simulated AM.

### 2.8 Statistical analysis

Statistical analysis was performed using GraphPad Prism 8.0 (Graph Pad Software, San Diego, CA, United States), and the data were reported as means ± standard deviation with the group size (n) representing the number of experiments. We then compared the means between the two groups using the unpaired Student’s t-test; comparisons of means among three or more groups were performed using one-way or two-way analysis of variance (ANOVA), followed by a *post hoc* test using the Tukey honestly significant difference method. The significance of the mean comparisons is represented using asterisks (*p < 0.05; **p < 0.01).

## 3 Results

### 3.1 Effects of Fe_2_O_3_ nanoparticles on the AM microstructure

**FIGURE 1:**
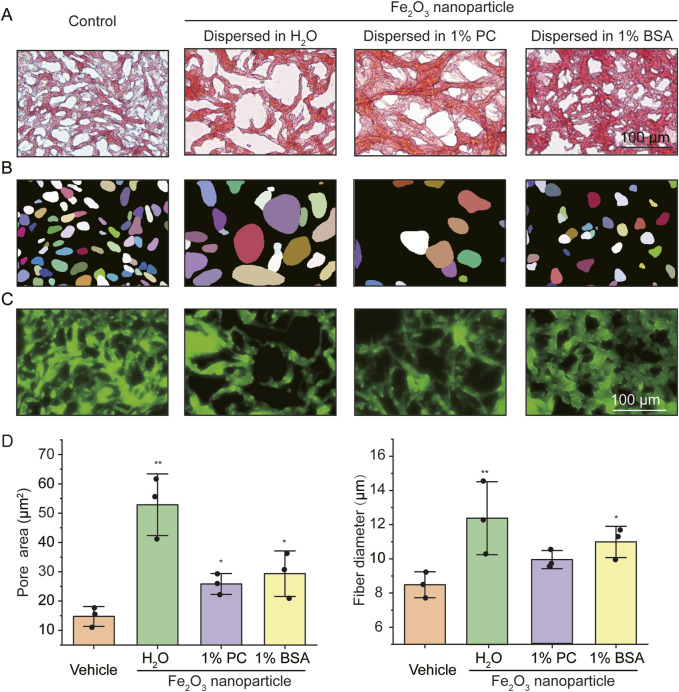
Microstructural characterization of the simulated asthmatic airway mucus (AM) with and without Fe_2_O_3_ nanoparticles. **(A–C)** Representative microscopic images of the simulated asthmatic AM (A: bright-field image of Ponceau S staining; B: digitized pores corresponding to A; C: immunofluorescence image of Mucin5A/C, scale bar = 100 μm). **(D)** Quantified pore areas and mucus fiber diameters under conditions corresponding to the images in **(A–C)**. PC: Phosphatidylcholine, BSA: bovine serum albumin, Control/Vehicle: without Fe_2_O_3_ nanoparticles; the data are presented as means ± SD, n = 3, *p < 0.05, **p < 0.01 vs. Vehicle.

The Fe_2_O_3_ nanoparticles used in this study were nearly spherical with an average size of ∼100 (70.73 ± 15.23) nm, as characterized by scanning electron microscopy (SEM) and dynamic light scattering (Supplementary Figure S1). The AM samples stained with Ponceau S (a total protein dye) showed a network structure with abundant pores for the proteins in AM under bright-field optical microscopy (Figure 1A); the presence of Fe_2_O_3_ nanoparticles appeared to disrupt the AM microstructure, which manifested as increased area of the pores. In addition, the disruptive effects of Fe_2_O_3_ nanoparticles on the microstructure were influenced by the medium in which the nanoparticles were dispersed. These pores were further digitally recognized using the StarDist program, as shown in Figure 1B. The simulated AM stained with Mucin5A/C-specific fluorescent antibody showed a cross-linked and entangled fiber structure that was also influenced by the nanoparticles and their dispersion medium (Figure 1C).

The disruptive effects of the Fe_2_O_3_ nanoparticles on the AM microstructure were quantified using the StarDist program in terms of the pore area of the protein structure and diameter of the mucin fiber. The results show that the average pore areas of the protein structures in the AM treated with vehicle (control) as well as Fe_2_O_3_ nanoparticles dispersed in H_2_O, 1% PC, and 1% BSA were 14.77 ± 0.75, 52.86 ± 10.51 (*p* = 0.003), 25.84 ± 3.56 (*p* = 0.017), and 29.34 ± 7.77 (*p* = 0.041) μm^2^ (Figure 1D left), while the average diameters of the Mucin5A/C fibers were 8.49 ± 0.76, 12.39 ± 2.13 (*p* = 0.040), 9.90 ± 0.53 (*p* = 0.056), and 10.98 ± 0.91 (*p* = 0.021) μm (Figure 1D right), respectively. The presence of the Fe_2_O_3_ nanoparticles also influenced the distributions of the pore areas and fiber diameters of the mucins within AM, indicating greater efficiency of the mucins for Fe_2_O_3_ nanoparticle dispersed in H_2_O, as shown in Supplementary Figure S2. Together, these findings suggest that the Fe_2_O_3_ nanoparticles disrupt the AM microstructure by inducing mucin aggregation, thereby resulting in increased pore sizes and fiber diameters in the AM than the control. Moreover, the Fe_2_O_3_ nanoparticles dispersed in H_2_O appeared to be more potent at disrupting the AM microstructure than those dispersed in 1% BSA and 1% PC, indicating that the effects of the nanoparticles were dependent on the dispersion medium.

### 3.2 Effects of Fe_2_O_3_ nanoparticles on the viscoelasticity of cured AM

**FIGURE 2:**
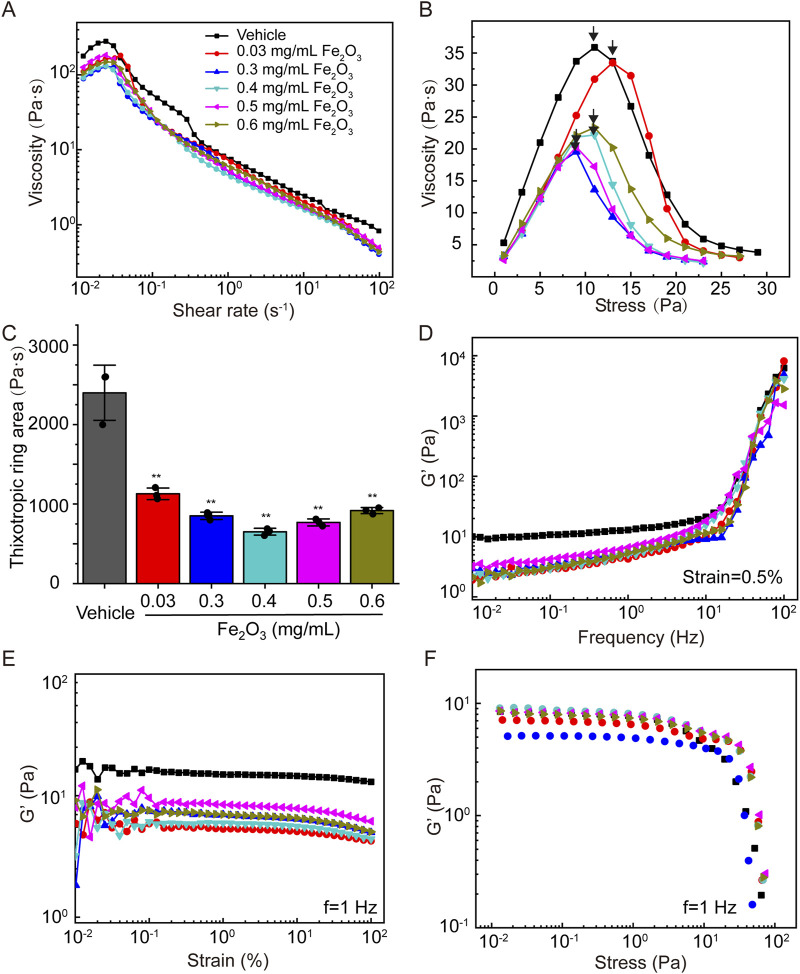
Effects of Fe_2_O_3_ nanoparticles on the rheological behaviors of cured AM. **(A)** Viscosity of AM as a function of shear rate under steady-state shearing. **(B)** Yield behaviors of AM as the viscosity was measured with shear stress ramping from 0 to 45 Pa over 5 min; the yield stress corresponding to the peak viscosity is indicated by the arrow. **(C)** Thixotropic ring area of the AM data presented as means ± SD, n = 3, **p < 0.01 vs. Vehicle. **(D–F)** Elastic modulus (G′) of AM as functions of the shear oscillation frequency (strain-controlled mode with a strain amplitude of 0.5% as the frequency is swept from 0.01 to 100 Hz), shear oscillation strain amplitude (frequency-controlled mode with a frequency of 1 Hz as the strain is swept from 0.01% to 100%), and shear oscillation stress amplitude (frequency-controlled mode with a frequency of 1 Hz as the stress is swept from 0.01 to 100 Pa).

Figure 2 shows the effects of the Fe_2_O_3_ nanoparticles on the rheological behaviors of cured AM. Specifically, in most of the cases, cured AM exhibited shear-thickening behavior, where the viscosity increased when the shear rate increased in a narrow range of very low shear rates (10^−2^ to 10^−1^ s^–1^), that changed to shear-thinning behavior as the shear rate increased further over a larger range from 10^−1^ to 10^2^ s^–1^. However, treating the cured AM with Fe_2_O_3_ nanoparticles seemed to decrease its apparent viscosity (Figure 2A); the Fe_2_O_3_ nanoparticles also caused significant reductions in the other viscoelastic properties, including yield stress, thixotropy, and storage modulus (G′) versus frequency/strain/stress (Figures 2B–F). For example, the yield stress (shear stress corresponding to the peak viscosity, as indicated by arrows) decreased from 29 Pa to 13 Pa when the AM was treated with 0.4 mg/mL of Fe_2_O_3_ nanoparticles compared to the control (Figure 2B). In addition, the storage modulus measured at a constant 0.5% strain magnitude with a swept oscillatory frequency from 10^−2^ to 10^2^ Hz had the typical characteristics of gel-like soft matter, whose internal microstructure is largely determined by the intermolecular forces (Figure 2D).

### 3.3 Effects of Fe_2_O_3_ nanoparticles on the viscoelasticity of curing AM

**FIGURE 3:**
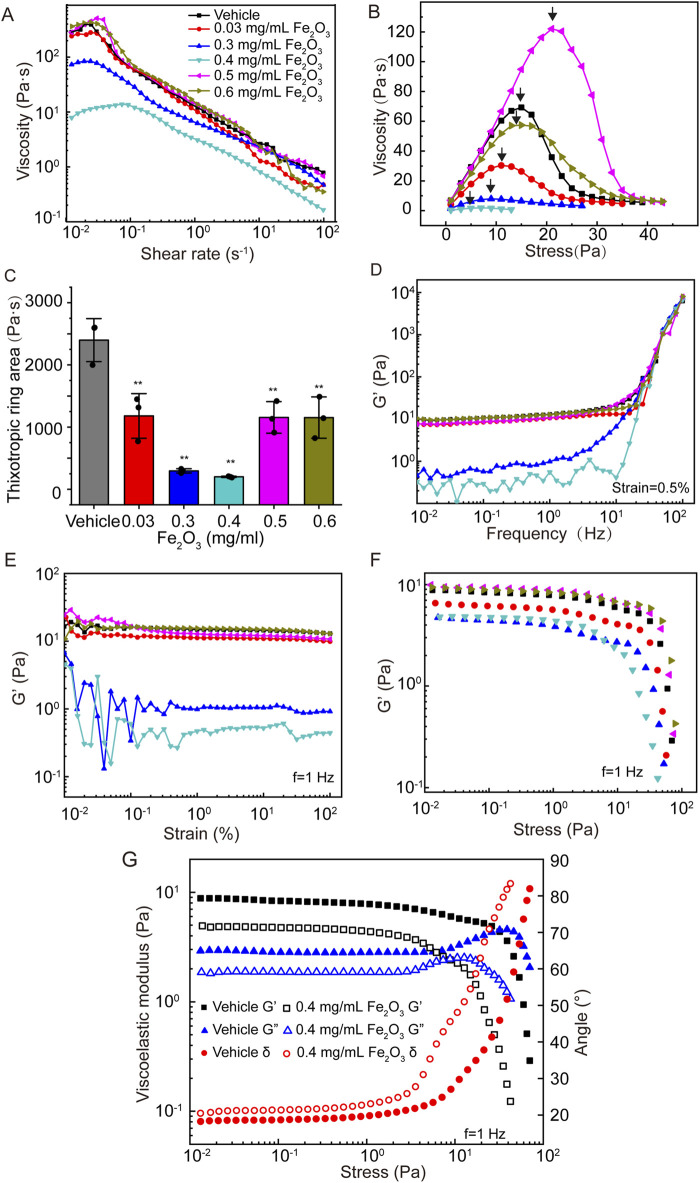
Effects of Fe_2_O_3_ nanoparticles on the rheologic behaviors of curing AM. **(A)** Viscosity of AM as a function of shear rate under steady-state shearing. **(B)** Yield behaviors of AM as the viscosity was measured with shear stress ramping from 0 to 45 Pa over 5 min; the yield stress corresponding to the peak viscosity is indicated by the arrow. **(C)** Thixotropic ring area of the AM data presented as means ± SD, n = 3, **p < 0.01 vs. Vehicle. **(D–F)** Elastic modulus (G′) of AM as functions of the shear oscillation frequency (strain-controlled mode with a strain amplitude of 0.5% as the frequency is swept from 0.01 to 100 Hz), shear oscillation strain amplitude (frequency-controlled mode with a frequency of 1 Hz as the strain is swept from 0.01% to 100%), and shear oscillation stress amplitude (frequency-controlled mode with a frequency of 1 Hz as the stress is swept from 0.01 to 100 Pa). **(G)** Elastic modulus (G′), viscous modulus (G″), and loss angle (θ) of AM as functions of the shear oscillation stress amplitude.

Figure 3 shows the effects of Fe_2_O_3_ nanoparticles on the rheological behaviors of curing AM. Unsurprisingly, treatment of curing AM with Fe_2_O_3_ nanoparticles resulted in reductions of all the viscoelastic properties similar to those for cured AM. Irrespective of treatment with Fe_2_O_3_ nanoparticles, the curing AM exhibited shear thickening as the shear rate increased from 10^−2^ to 10^−1^ s^–1^ that changed to shear thinning as the shear rate further increased from 10^−1^ to 10^2^ s^–1^. Nevertheless, treatment with Fe_2_O_3_ nanoparticles, especially at a concentration of 0.4 mg/mL, largely decreased the apparent viscosity across the entire range of shear rates (Figure 3A). For curing AM, treatment with Fe_2_O_3_ nanoparticles at concentrations of 0.03, 0.3, 0.4, 0.5, and 0.6 mg/mL caused changes in the yield stress from 15.00 Pa for the control to 11.21, 0.99, 0.70, 21.12, and 15.02 Pa, respectively, while the thixotropic ring area also changed similarly. Again, Fe_2_O_3_ nanoparticles at 0.4 mg/mL appeared to be the most potent at reducing the yield stress and thixotropy (Figures 3B,C). The frequency dependence of the storage modulus of curing AM without Fe_2_O_3_ nanoparticles was similar to that of cured AM, indicating typical soft-matter dynamics behavior. Nevertheless, the effects of the Fe_2_O_3_ nanoparticles on this dynamic behavior as well as on the strain/stress dependence of G′ for curing AM were much greater than those of cured AM. Once again, a concentration of 0.4 mg/mL of Fe_2_O_3_ nanoparticles appeared to be the most potent for reducing the storage moduli across the chosen ranges of frequency, strain, and stress values (Figures 3D–F).

It should be noted that the stress amplitude oscillation scanning curve of the storage modulus (G′) could be used to explore the non-linear behaviors of mucus. As shown in Figure 3F, the G’ of AM was almost invariable at small stress values (<1 Pa). As the stress increased, this value decreased gradually and crossed over at approximately 50 Pa, suggesting disruption of the mucus microstructure and establishment of non-linear viscoelastic behavior. For the control of curing AM, the LVE behavior was limited to stress amplitudes below 67 Pa. In contrast, curing AM treated with Fe_2_O_3_ nanoparticles was limited to far lower stress amplitudes to ensure linear viscoelasticity, e. g., 10 Pa at 0.4 mg/mL. Furthermore, as shown in Figure 3G, the G’ values were invariably greater than G” in all cases at small stress values (<1 Pa), suggesting that the storage modulus dominated loss modulus or classical elastic behaviors. As the stress levels increased, G′ and G″ gradually crossed over; thereafter, the loss modulus dominated the storage modulus, indicating a fluid-like state of the AM. We also noted that the curing AM treated with 0.4 mg/mL of Fe_2_O_3_ nanoparticles had a lower stress level of 2 Pa for crossover compared to 30 Pa for the control. These data indicate that the Fe_2_O_3_ nanoparticles were able to fluidize the curing AM, which is consistent with their ability to disrupt the microstructure of AM.

### 3.4 Effects of surface properties of Fe_2_O_3_ nanoparticles on the viscoelasticity of curing AM

**FIGURE 4:**
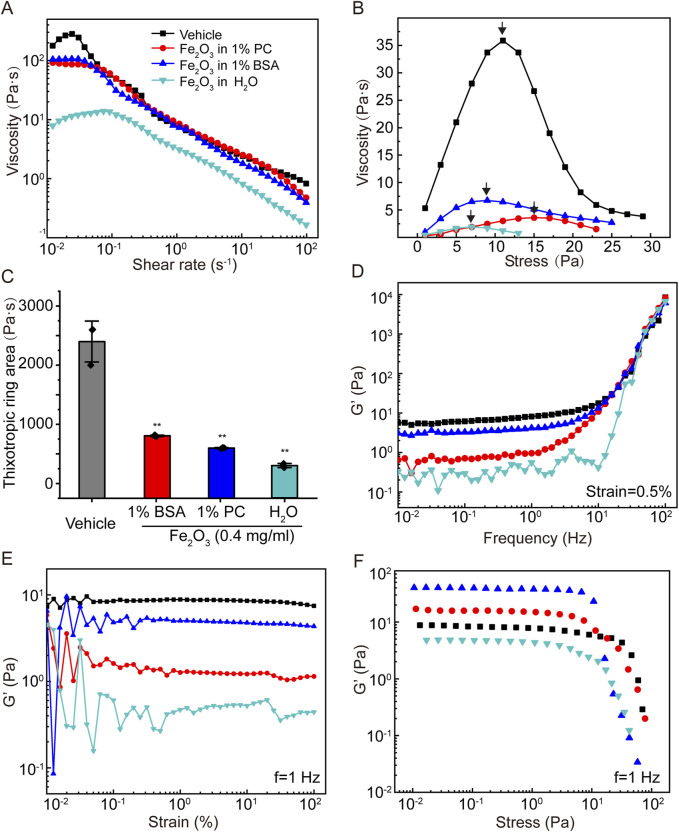
Effects of the surface chemical properties of Fe_2_O_3_ nanoparticles on the rheological behaviors of AM. **(A)** Viscosity of AM as a function of shear rate under steady-state shearing. **(B)** Yield behaviors of AM as the viscosity was measured with shear stress ramping from 0 to 45 Pa over 5 min; the yield stress corresponding to the peak viscosity is indicated by the arrow. **(C)** Thixotropic ring area of the AM data presented as means ± SD, n = 3, **p < 0.01 vs. Vehicle. **(D–F)** Elastic modulus (G′) of AM as functions of the shear oscillation frequency (strain-controlled mode with a strain amplitude of 0.5% as the frequency is swept from 0.01 to 100 Hz), strain amplitude (frequency-controlled mode with a frequency of 1 Hz as the strain is swept from 0.01% to 100%), and stress amplitude (frequency-controlled mode with a frequency of 1 Hz as the stress is swept from 0.01 to 100 Pa).

The effects of the surface properties of the nanoparticles on the viscoelasticity of curing AM were investigated using 0.4 mg/mL of Fe_2_O_3_ nanoparticles dispersed in H_2_O, 1% BSA, or 1% PC. To examine whether the Fe_2_O_3_ nanoparticles adsorbed lipids or albumins onto their surfaces when dispersed in PC or BSA, we stained the nanoparticles with dio or FITC-BSA, respectively, and obtained images with bright-field and immunofluorescence microscopy. The results shown in Supplementary Figure S3 indicate that the Fe_2_O_3_ nanoparticles indeed adsorbed albumins and lipids on their surfaces when dispersed in BSA (1%) and PC (1%), respectively. The results shown in Figure 4 indicate that the Fe_2_O_3_ nanoparticles dispersed in 1% BSA, 1% PC, or H_2_O and having different surface properties could generally alter the viscoelastic behaviors of curing AM. However, these alterations were variably dependent on whether the nanoparticles were dispersed in 1% BSA, 1% PC, or H_2_O. In fact, compared to 1% BAS and 1% PC, H_2_O was found to be the optimal dispersion medium in which the Fe_2_O_3_ nanoparticles caused the largest changes in all viscoelastic properties, indicating that the surface properties may be important for the efficacy with which the Fe_2_O_3_ nanoparticles reduce the viscoelasticity of AM.

### 3.5 Effects of Fe_2_O_3_ nanoparticles or chymotrypsin on the viscoelasticity of cured AM

**FIGURE 5:**
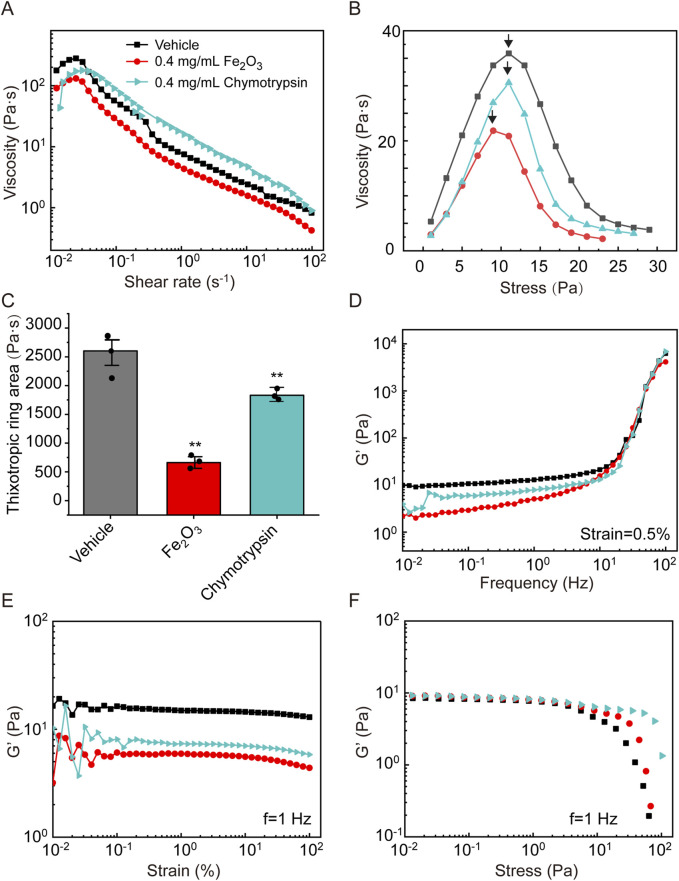
Effects of Fe_2_O_3_ nanoparticles and the conventional mucolytic chymotrypsin on the rheological behaviors of AM. **(A)** Viscosity of AM as a function of shear rate under steady-state shearing. **(B)** Yield behaviors of AM as the viscosity was measured with shear stress ramping from 0 to 45 Pa over 5 min; the yield stress corresponding to the peak viscosity is indicated by the arrow. **(C)** Thixotropic ring area of the AM data presented as means ± SD, n = 3, **p < 0.01 vs. Vehicle. **(D–F)** Elastic modulus (G′) of AM as functions of the shear oscillation frequency (strain-controlled mode with a strain amplitude of 0.5% as the frequency is swept from 0.01 to 100 Hz), strain amplitude (frequency-controlled mode with a frequency of 1 Hz as the strain is swept from 0.01% to 100%), and stress amplitude (frequency-controlled mode with a frequency of 1 Hz as the stress is swept from 0.01 to 100 Pa).

To evaluate the mucolytic potential of the Fe_2_O_3_ nanoparticles, we compared their effects on the viscoelasticity of cured AM with those of the conventional mucolytic chymotrypsin that is widely used in clinical practice. The cured AM was treated with either 0.4 mg/mL of Fe_2_O_3_ nanoparticles dispersed in H_2_O or 0.4 mg/mL of chymotrypsin and evaluated for the viscoelastic properties. The results indicate that both Fe_2_O_3_ nanoparticles and chymotrypsin were able to lower the apparent viscosity, yield stress, and thixotropy (Figures 5A–C) as well as the storage modulus of the cured AM over the chosen ranges of frequency, strain, and stress values (Figures 5D–F). Interestingly, the Fe_2_O_3_ nanoparticles appeared to reduce all viscoelastic properties to a greater extent, especially thixotropy, compared to chymotrypsin at the same concentration. This suggests that the Fe_2_O_3_ nanoparticles may be more potent than conventional mucolytics for assisting with AM clearance. Furthermore, identical rheological experiments conducted with real mucus samples collected from asthmatic patients show very similar effects of the Fe_2_O_3_ nanoparticles on the mucus viscosity, yield stress, thixotropy, and storage modulus over the chosen ranges of frequency, strain, and stress values (Supplementary Figure S4).

## 4 Discussion

Based on evaluations of the microstructure and macrorheological behaviors of simulated asthmatic AM treated with Fe_2_O_3_ nanoparticles either during or after the curing process, our primary finding is that the Fe_2_O_3_ nanoparticles could disrupt the microstructure mainly by increasing the pore size and thereby reducing the viscoelastic properties of both the curing and cured AM, including reduced viscosity, yield stress, and thixotropy with enhanced fluid-like state depending on the nanoparticle concentration and their surface properties. In addition, Fe_2_O_3_ nanoparticles dispersed at 0.4 mg/mL in H_2_O were highly potent at altering the magnitudes of the rheological properties of AM, even exceeding those achieved with 0.4 mg/mL of the conventional mucolytic chymotrypsin. These findings suggest that Fe_2_O_3_ nanoparticles can be used as expectorant agents for AM clearance therapy.

Human AM is a non-Newtonian fluid exhibiting complex rheological behaviors, such as shear thinning/thickening, thixotropy, and dynamic viscoelasticity, which are primarily the results of entanglement and cross-linking of the internal polymeric structures of proteins like mucins. Therefore, it is very important to study the rheological properties of AM to understand the pathologies of respiratory complications associated with AM embolism and develop a more effective method for AM clearance in clinical practice. However, it is usually difficult to obtain human AM in quantities sufficient for conventional bulk rheometric analyses. To overcome this limitation, mucin-based protein solutions that can simulate AM in diseased states such as asthma are widely used in various studies, including rheological studies. Thus, we adapted one such preparation of simulated asthmatic AM according to reports from literature. In this study, the prepared AM displayed rheological behaviors similar to those of simulated and real asthmatic AM in published reports (Jory et al., 2022; Cone, 2009). The AM prepared in this study also exhibited a power law behavior, which is in agreement with the widely recognized behaviors of soft matter like cells and protein gels (Jory et al., 2022; Deng et al., 2006; Yang et al., 2018; Vasquez et al., 2014; Ewoldt et al., 2008).

We also hypothesized that Fe_2_O_3_ nanoparticles could be used to alter the rheological behaviors of AM considering their unique surface physical and chemical properties (Montiel Schneider et al., 2022). Our test results showed that the Fe_2_O_3_ nanoparticles indeed reduced the viscosity and yield stress of both curing and cured AM, but the effects were more potent in the former. These properties may be used to promote mucus clearance through coughing, sneezing, and percussive therapies (Yi et al., 2021; Kim et al., 2020; Lauga and Hosoi, 2006; Montenegro-Johnson et al., 2013; Evans and Koo, 2009; Grotberg, 2001; Grotberg, 2011). It is unusual that the addition of Fe_2_O_3_ nanoparticles would decrease the viscosity of AM; as such, the largest decrease occurred at 0.4 mg/mL instead of decreasing monotonically with the Fe_2_O_3_ nanoparticle concentration. Such peculiar effects of the Fe_2_O_3_ nanoparticles on mucus rheology cannot be predicted from Einstein’s canonical theory, and the underlying mechanisms remain elusive. However, this phenomenon likely reflects the effects of adsorption of the mucus contents onto the Fe_2_O_3_ nanoparticles; this depletes the mucin polymers to prevent cross-linking and fiber formation while reducing the solution viscosity; it also has the effect of increasing the free volume of the particles and polymers at low concentrations of Fe_2_O_3_ nanoparticles (Mackay et al., 2003). All of these effects could contribute as underlying mechanisms for disrupting the microstructure and altering the rheological properties of mucus by Fe_2_O_3_ nanoparticles. However, at high concentrations, the interactions between the Fe_2_O_3_ nanoparticles and mucin fibers may be enhanced to overcome the nanoscale effects, resulting in an increase in the solution viscosity as predicted by Einstein’s theory.

Microscopic examinations of the AM treated with and without Fe_2_O_3_ nanoparticles revealed that the presence of the nanoparticles caused marked disruption in the AM microstructure, which was probably responsible for the changes in the viscoelastic properties of the AM. Our optical observations revealed a mesh architecture of the AM, which is consistent with the structure of the mucin network within sputum (Ghanem et al., 2021). The microstructural alterations in the AM owing to the presence of the Fe_2_O_3_ nanoparticles manifested as increased pore sizes and fiber diameters of the protein meshwork, which is mainly composed of cross-linked and entangled mucin polymeric fibers. The pore sizes were observed with optical microscopy and appeared to be much larger than those observed with SEM in human AM (Schuster et al., 2013; Sanders et al., 2000); this discrepancy may be attributed to dehydration of the mucus sample during the vacuum treatment required before SEM. The mechanisms by which the Fe_2_O_3_ nanoparticles disrupt the mucus microstructure are not fully understood, although these may be largely attributed to the surface interactions with mucins, such as adsorption, since nanoparticles have large surface-to-volume ratios and high surface energies; these properties have been reported to produce conformational changes in polymers and affect viscosity via interactions between the particles and polymers, including adsorption, depletion, and grafting (Negrini et al., 2017; Goodwin et al., 2013). For example, mucins are glycoproteins containing many oligosaccharide chains that are terminated by negatively charged carboxyl groups like sialic acid (Li et al., 2013; Sellers et al., 1988). These negative charges may further enhance the adsorption of mucins by the Fe_2_O_3_ nanoparticles and lead to aggregation of mucins, with consequent disruption of the mucus microstructure. The interactions between the Fe_2_O_3_ nanoparticles and mucins can also lead to other molecular events, such as structural reorganization and conformational disruption, which could contribute to changes in the mucus microstructure and rheology. In this work, we demonstrated that Fe_2_O_3_ nanoparticles dispersed in 1% BSA or 1% PC were less potent at changing the mucus microstructure and rheology compared to those dispersed in pure water (H_2_O); this finding supports the idea that the surface properties of the Fe_2_O_3_ nanoparticles play essential roles in mediating the microstructural and macrorheological changes in mucus.

It was apparent from our evaluations that different kinds of surface modifications change the density and distribution of the surface charges and binding structures of the nanoparticles. Subsequently, when differently premodified nanoparticles are added to the mucus, their interactions with the mucus components as well as consequent effects on the structure and rheology of the mucus are expected to vary, as observed in our experiments. It is also predicted that nanoparticles with albumins or lipids adsorbed on their surfaces may be less potent at occupying the cross-linking sites of the mucin polymers and hence less effective at altering the microstructure and rheology of mucus; this was confirmed by fluorescence microscopy. The extent to which mucus components could be adsorbed onto the surface of the Fe_2_O_3_ nanoparticles remains unknown and needs to be thoroughly investigated in the future to fully understand the effects of surface adsorption on the rheological properties of mucus (Negrini et al., 2017).

Our results show that the storage and loss moduli of AM are highly dependent on the frequency and stress; in the frequency range of 0.01–10 Hz and stress range of 0.01–1 Pa, the storage modulus was almost always greater than the loss modulus, indicating that AM treated both with and without Fe_2_O_3_ nanoparticles exhibited a solid-like behavior. Such elastic behaviors of the AM agreed well with those observed previously for various mucins, which could be explained by the cross-linking of mucins to form fiber-like network structures in mucus (Vasquez et al., 2016). The frequency range was selected for its physiological and pathological relevance since mucus is known to experience an oscillatory shear of ∼6 rad s^–1^ during extrusion from the glands, ∼60 rad s^–1^ during ciliary beating, and >60 rad s^–1^ during coughing, high-frequency ventilation, or percussive therapy (Vasquez et al., 2016). Thus, the frequency dependences of the storage and loss moduli provide essential information for understanding mucus transport.

As a conventional mucolytic drug used widely in clinical practice to assist AM clearance in the treatment of airway diseases like asthma, chymotrypsin was used in this study at 0.4 mg/mL and found to reduce the magnitudes of the rheological properties of the simulated asthmatic AM in most cases. However, our data demonstrated that Fe_2_O_3_ nanoparticles at the same concentration could reduce the magnitudes of the rheological properties of mucus to a greater extent than chymotrypsin. In addition to their ability to reduce the viscoelasticity of AM, Fe_2_O_3_ nanoparticles are naturally biocompatible because iron is an essential element required by the human body. In fact, Fe_2_O_3_ nanoparticles have already been approved by the United States Food and Drug Administration for other clinical applications like imaging enhancement (Montiel Schneider et al., 2022) and treatment of iron-deficiency anemia in adult patients with chronic kidney disease (Mitchell et al., 2021). Considering the intrinsic safety, cost benefits, and ability to reduce AM viscoelasticity, it is reasonable to expect satisfactory outcomes in future clinical trials on validating clinical AM clearance applications with Fe_2_O_3_ nanoparticles as alternatives to conventional chemical mucolytics (Bobo et al., 2016).

It should be noted that only the macrorheological properties of mucus were assessed in this study as they mainly determine the mucus bulk fluidity and are thus more relevant to mucus transport by the mucociliary system as well as cough-induced mucus clearance despite the potential overestimation of the true bulk viscosity of mucus (Hill et al., 2022; Kavishvar and Ramachandran, 2023). With regard to drug delivery or pollutant invasion through the mucus layer, it is more relevant to use microrheological techniques to probe the viscoelastic properties of mucus at submicron scales, such as particle tracking microrheology or microbead magnetic systems (Lai et al., 2009b). We also note that the microrheological properties of AM could possibly differ from the macrorheological properties presented herein. It is worth noting that the present findings from simulated asthmatic AM and limited numbers of real mucus samples from asthmatic patients need to be further investigated in the future through *in vitro* and *in vivo* studies along with real human AM of both healthy and asthmatic subjects. Furthermore, the underlying mechanisms of the surface-mediated effects of Fe_2_O_3_ nanoparticles on the microstructure and microrheological behaviors of AM need to be elucidated.

## 5 Conclusion

Fe_2_O_3_ nanoparticles can induce surface-mediated mucin aggregation and reduce the viscoelastic properties like viscosity, yield stress, and storage/loss moduli, thus increasing the fluidity of simulated asthmatic AM. The mucolytic potency of the Fe_2_O_3_ nanoparticles may even be greater than that of conventional mucolytics like chymotrypsin. Together, these findings suggest that Fe_2_O_3_ nanoparticles may have great potential in the future development of novel expectorant agents for AM clearance applications.

## Data Availability

The original contributions presented in this study are included in the article/Supplementary Material, and any further inquiries may be directed to the corresponding authors.

## Supplementary material

SUPPLEMENTARY FIGURE S1 Morphology and size distribution of the Fe_2_O_3_ nanoparticles evaluated via **(A)** scanning electron microscopy and **(B)** dynamic light scattering.

SUPPLEMENTARY FIGURE S2 Histograms showing the distributions of **(A)** pore area and **(B)** mucus fiber diameter within airway mucus (AM) with and without Fe_2_O_3_ nanoparticles dispersed in H_2_O, 1% phosphatidylcholine (PC), or 1% bovine serum albumin (BSA).

SUPPLEMENTARY FIGURE S3 Representative immunofluorescence images showing the adsorption of lipids or albumins on the surfaces of the Fe_2_O_3_ nanoparticles dispersed in 1% BSA or 1% PC, respectively. Scale bar = 100 μm.

SUPPLEMENTARY FIGURE S4 Effects of Fe_2_O_3_ nanoparticles and the conventional mucolytic chymotrypsin on the rheological behaviors of mucus from asthmatic patients. **(A)** Viscosity as a function of shear rate under steady-state shearing. **(B)** Yield behavior as viscosity was measured with shear stress ramping from 0 to 45 Pa over 5 min; the yield stress corresponding to the peak viscosity is indicated by the arrow. **(C)** Thixotropic ring area with the data presented as mean ± SD for n = 3; ***p* < 0.01 vs. control group. **(D–F)** Elastic modulus (G′) as functions of the shear oscillation frequency (strain-controlled mode with a strain amplitude of 0.5% as the frequency is swept from 0.01 to 100 Hz), strain amplitude (frequency-controlled mode with a frequency of 1 Hz as the strain is swept from 0.01% to 100%), and stress amplitude (frequency-controlled mode with a frequency of 1 Hz as the stress is swept from 0.01 to 100 Pa).

## Abbreviations

AM, airway mucus; ANOVA, analysis of variance; BSA, bovine serum albumin; G′, elastic modulus; G″, viscous modulus; LVE, linear viscoelastic; PC, phosphatidylcholine.
