# Supplementary figures and images for: Fe2O3 nanoparticles disrupt microstructure and reduce the viscoelasticity of simulated asthma airway mucus for potential airway mucus clearance applications
Source: Front Physiol. 2025 Jun 30;16:1566716. doi: 10.3389/fphys.2025.1566716 (PMC12256507; doi:10.3389/fphys.2025.1566716)

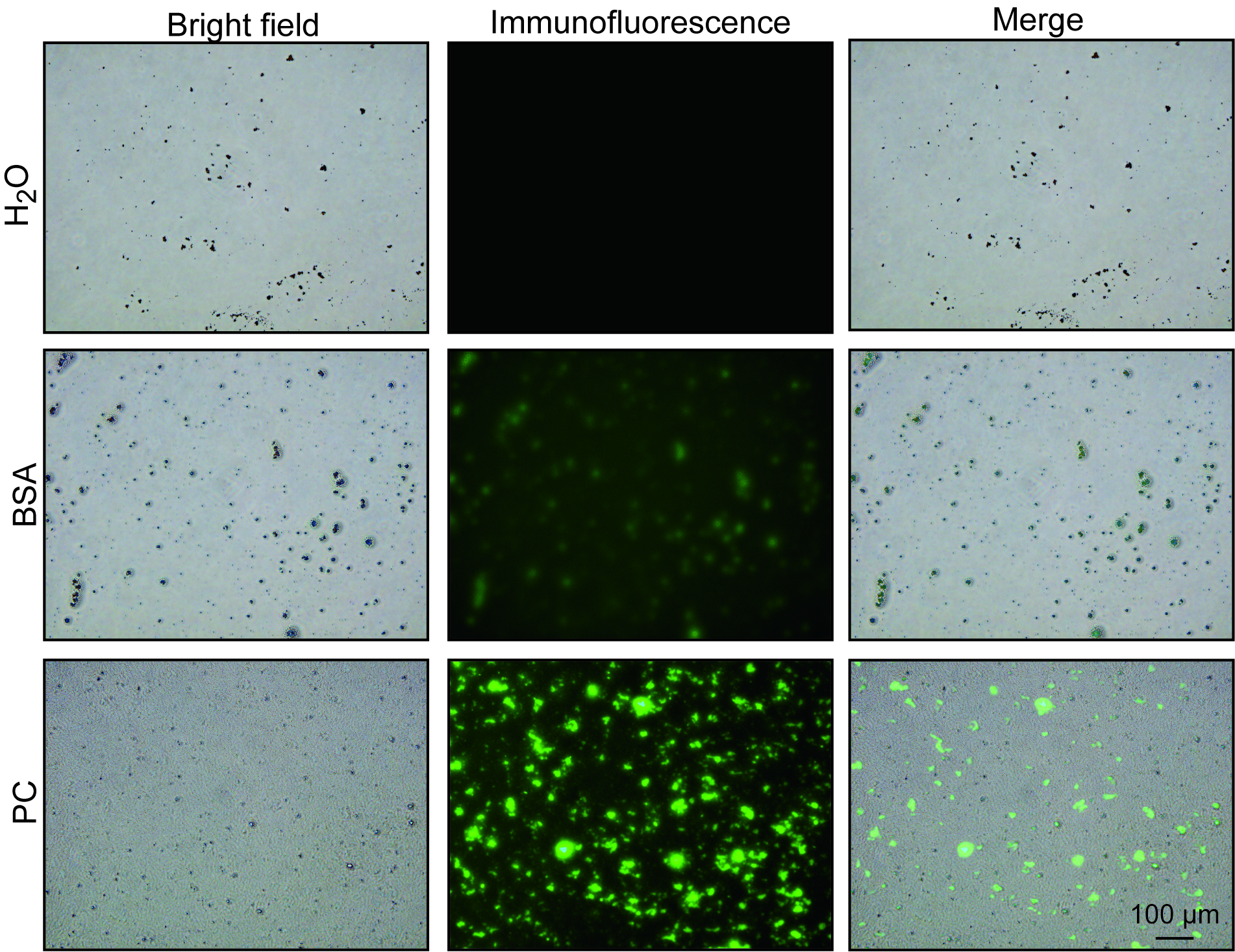

Supplement: Supplementary file 1 [file Image3.tif]

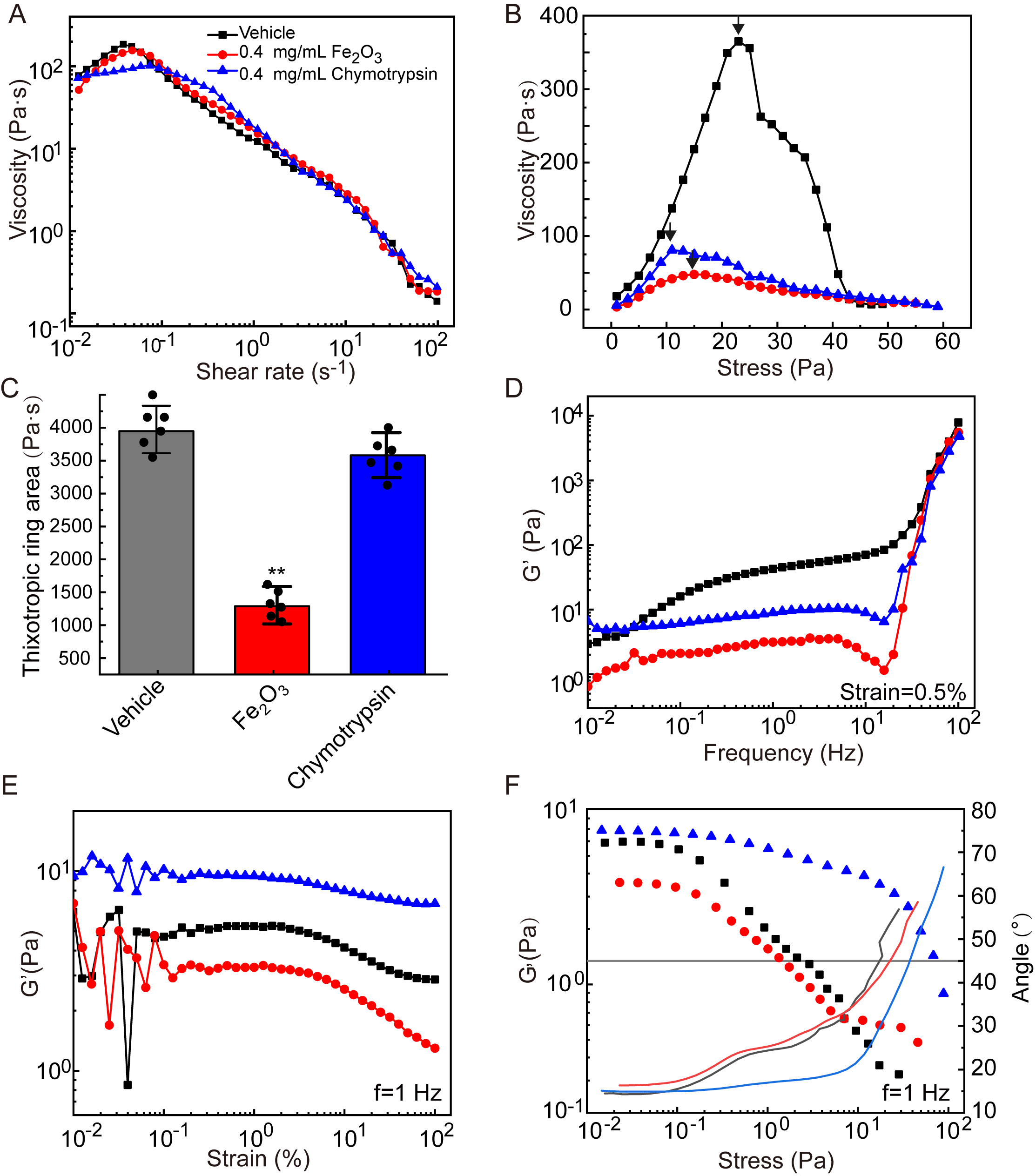

Supplement: Supplementary file 2 [file Image4.tif]

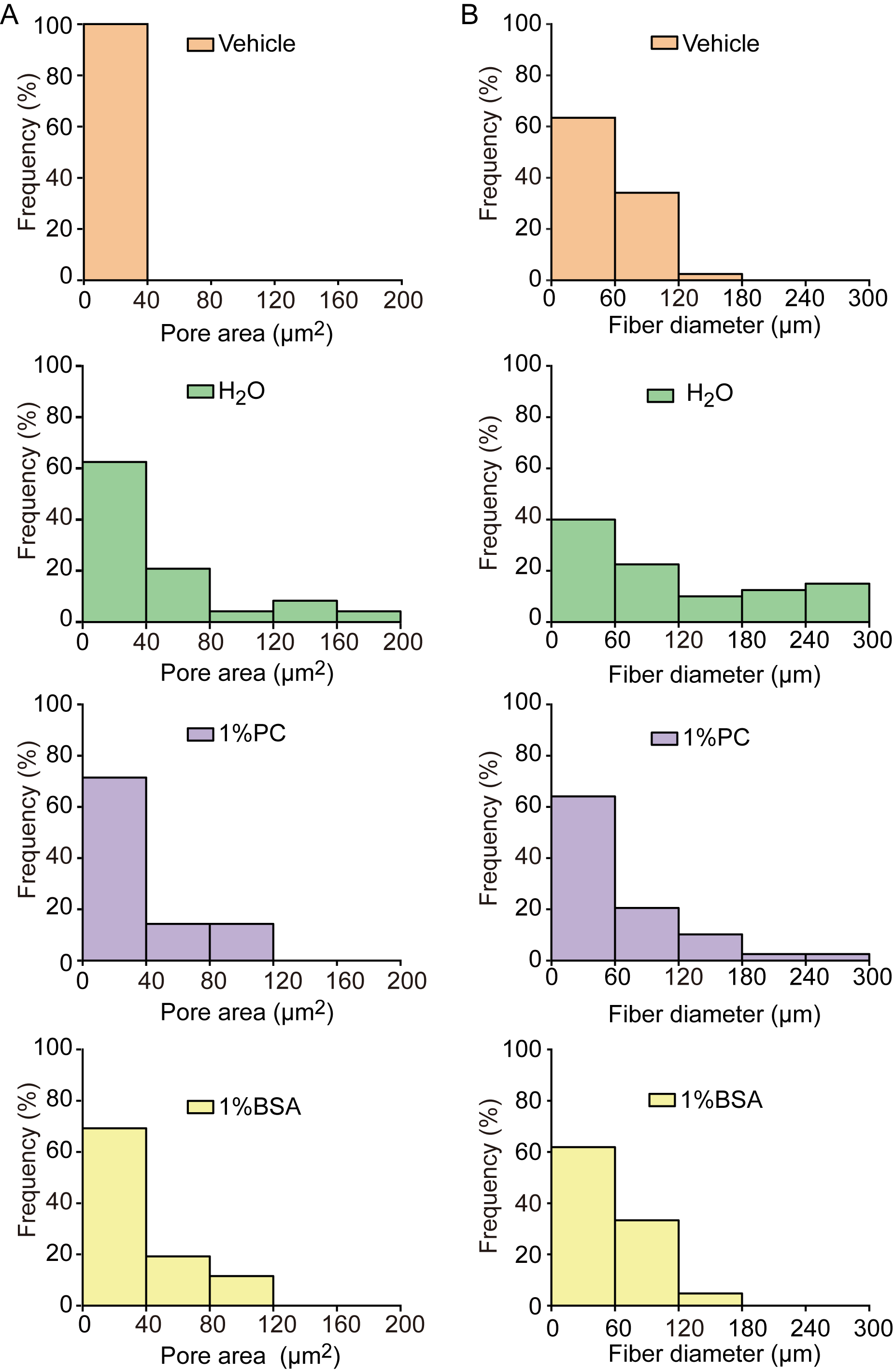

Supplement: Supplementary file 3 [file Image2.tif]

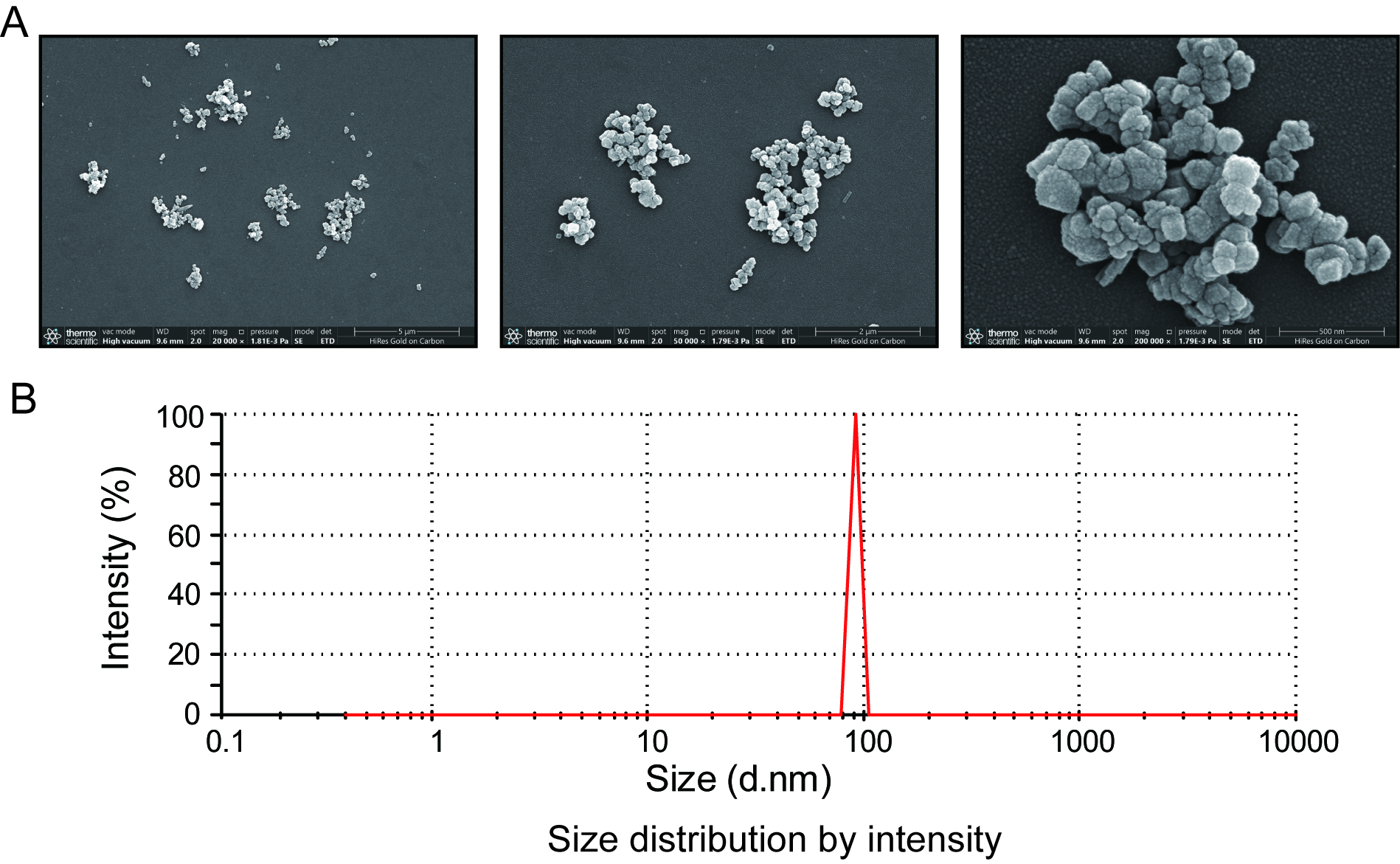

Supplement: Supplementary file 4 [file Image1.tif]
